# A longitudinal study of COVID-19 preventive behavior fatigue in Hong Kong: a city with previous pandemic experience

**DOI:** 10.1186/s12889-023-15257-y

**Published:** 2023-03-31

**Authors:** Jean H. Kim, Kin On Kwok, Zhe Huang, Paul Kwok-ming Poon, Kevin Kei Ching Hung, Samuel Yeung Shan Wong, Emily Ying Yang Chan

**Affiliations:** 1 JC School of Public Health and Primary Care, The Chinese University of Hong Kong, Hong Kong, China; 2 Collaborating Centre for Oxford University and CUHK for Disaster and Medical Humanitarian Response (CCOUC), The Chinese University of Hong Kong, Hong Kong SAR, China; 3 GX Foundation, Hong Kong, China; 4 Accident & Emergency Medicine Academic Unit, Prince of Wales Hospital, The Chinese University of Hong Kong, Hong Kong, China

**Keywords:** COVID-19, Epidemiology, Health behaviors, China, Infection control

## Abstract

**Background:**

In addition to high vaccination levels, COVID-19 control requires uptake and continued adherence to personal hygiene and social distancing behaviors. It is unclear whether residents of a city with successive experience in worldwide pandemics such as SARS, would quickly adopt and maintain preventive behaviors.

**Methods:**

A population-based, longitudinal telephone survey was conducted between in first local wave of the COVID-19 pandemic (April 2020) and third local wave (December 2020) (n = 403). The study examined factors associated with personal hygiene and social distancing behavior fatigue, as measured by reduced adherence.

**Results:**

Over 9 months, face mask use increased (96.5–100%, p < 0.001). Although habitual hand hygiene remained unchanged (92.0%), blue collar workers and non-working individuals showed higher risk of hand hygiene fatigue. There was a decline (p < 0.05) in avoidance of social gatherings (81.1 to 70.7%), avoidance of public places (52.9–27.5%) and avoidance of international travel (81.9–77.4%) even with rising caseloads. Lowered perception of COVID-19 disease severity was associated with decreased avoidance of social gatherings and public places while lower education was associated with decline in avoidance of social gatherings.

**Conclusion:**

Even in regions with past pandemic experience, maintaining social distancing behaviors during a protracted pandemic remains a major public health challenge.

## Background

Since its emergence in late 2019, the Coronavirus disease 2019 (COVID-19) pandemic has placed enormous strain on national health systems worldwide. Globally, over 670 million reported cases and nearly 6.83 million deaths have been attributed to this coronavirus as of February, 2023 [1]. Along with the personal and community protection from vaccine-induced immunity, reducing person-to-person transmission through personal preventive behaviors will continue to be key factors in COVID-19 control. However, levels of compliance with these hygiene behaviors such as wearing masks has varied widely across populations. In certain regions such as Hong Kong it was noted that virtually all residents quickly adopted the use of face masks both indoors and outdoors in the early epidemic phase [2], while in other countries such as in the United States there has been continued resistance to mask-wearing mandates despite rising daily caseloads [3–5]. Moreover, implementation of social distancing measures across countries of varying durations and levels of stringency have been increasingly facing resistance from the public as well as business owners [6, 7]. Even in populations with high adoption of personal hygiene and social distancing measures, behavioral fatigue in adhering to these practices may represents a major public health challenge [8]. Regions that had shown successful containment of COVID-19 early in the pandemic such as Taiwan, Vietnam and South Korea have subsequently reported surges in COVID-19 cases [9].

**Fig. 1:**
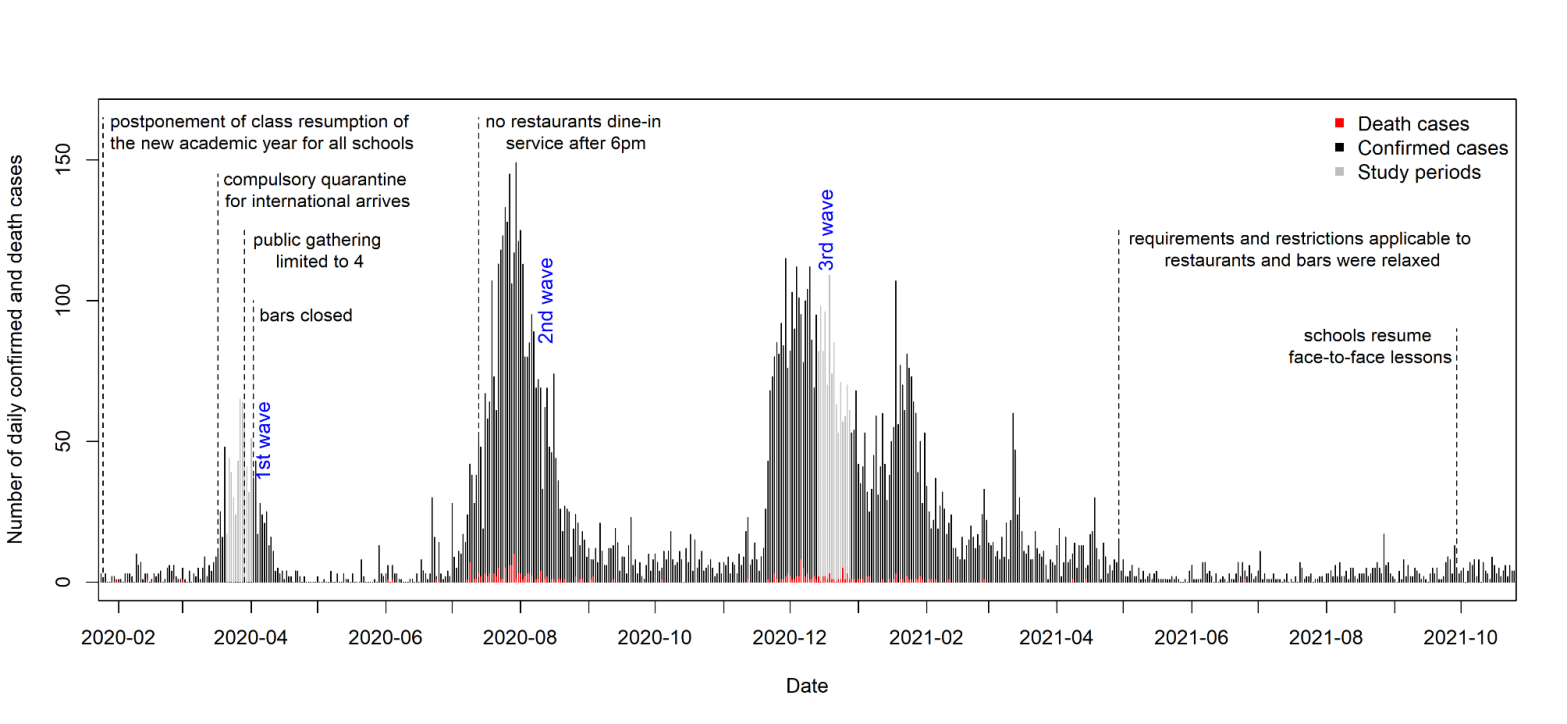
Timeline of the COVID-19 outbreaks and government actions in Hong Kong January 2020-December 2021

Hong Kong is a major travel hub at the juncture of mainland China and South-East Asia. Unlike many regions of the world, however, Hong Kong has experienced many previous major outbreaks in recent decades such as avian influenza A (H5N1) first in 1997 and then periodically since 2002, followed by SARS in 2003 and pandemic influenza in 2009. Prior sensitization to these outbreaks was believed to have significantly contributed to voluntarily uptake of preventative behaviors such as face mask use and avoidance of crowded public areas during the current epidemic [10–13]. Since January 2020, there have been successive COVID-19 epidemic waves in Hong Kong [14]. Health authorities have sought to minimize transmission of the virus through promotion of vaccine uptake, implementation of mandatory quarantines for international arrivals and promotion of various personal hygiene and social distancing measures [15]. This was later followed by closure of schools, recommendations to work from home, closure of bars and reduced dine-in hours at restaurants (see Fig. 1) [16]. Similar to studies conducted elsewhere [17–20], a past local serial cross-sectional study noted that while adherence to mask wearing and handwashing remained high throughout the early pandemic, social distancing decreased over time [2]. A longitudinal study can further examine individual factors associated with reduced adherence with infection prevention practices. Informed by the Health Belief Model, this study will also examine changing perceptions of risk (perceived transmissibility of the virus and perceived severity of COVID-19 disease) as well as changing perceptions of the benefits of these behaviors as possible predictors of behavioral fatigue [20]. Although COVID-19 has posed unique and difficult challenges [21–23], past studies conducted in Hong Kong had noted that perceptions of susceptibility and illness severity decreased over time for diseases such as SARS and H5N1 avian influenza [24, 25]. The primary objective of this study is to examine the uptake of various infection prevention measures of the population and to determine the levels of behavioral fatigue for each of these measures over a 9-month follow-up period. The study also seeks to examine the predictors of behavioral fatigue in this population.

The objectives of this study are to examine variations of fatigue in adopting the COVID-19 preventive practices among adults in two successive waves of the local epidemic in March and December 2020 in which 766 and 8725 local cases had been reported, respectively. The results of this study may provide insights about behavioral fatigue for other regions of the world that will experience future pandemics and will provide insights for those countries that remain high-risk COVID-19 regions in the long-term. This study sheds light on possible patterns of pandemic fatigue to other populations with outbreak experience in previous SARS and avian influenza epidemic to inform their future policies in the ongoing COVID-19 pandemic.

## Methods

### Study design and sampling

Using random digit dialing, a baseline telephone survey was conducted during the first local epidemic wave March 2020 (survey conducted from 22nd March to 1st April 2020). The target population was Cantonese-speaking Hong Kong residents aged 18 years old or above, including individuals holding work or study visas. Phone calls were made in the evenings on weekdays, and mornings and afternoons on weekends to prevent overrepresentation of the unemployed. The household members with birthday closest to the interview date were recruited in the baseline survey (‘last birthday’ method [26]). After the purpose of the survey was explained and assurance of confidentiality of response were made to potential respondents, oral consent was obtained from participants. The study followed up the respondents in nine months during the third epidemic wave in December 2020 (survey conducted from 15th to 29th December 2020) (see Fig. 1).

### Data collection and data management

In the baseline recruitment, subjects were asked to provide information of their sociodemographic and background characteristics. Respondents were also asked channels to access COVID-19 news: television, internet, smartphone applications (Yes/No). At both baseline and follow-up recruitment participants were asked how frequently they practiced five key infection prevention measures. The baseline and follow-up recruitment was done amid the first and third epidemic wave respectively. During both recruitment periods, the local government had tightened social distancing measures on gathering [27], travel restrictions [28], quarantine of inbound travelers [29], closure of restaurant [30], etc. The order of magnitude of case number were similar from 24 to 65 cases per day during the baseline recruitment and from 53 to 109 cases per day during the follow-up recruitment [31]. These behaviors included two hygiene measures (wearing a mask, frequent handwashing) and three types of social distancing measure (avoidance of public transport, avoidance of social gatherings, and avoidance of international travel) (see Table 1). For each preventive behavior, the level of adoption was assessed by questions using four-point Likert scale responses (1 = Always, at every opportunity, 2 = usually practiced/most of the time,3 = occasionally practiced/slightly above pre-pandemic levels, 4 = never practiced/same as pre-pandemic levels). To assess behavioral fatigue between baseline and follow-up, respondents who reported “All the time” or “Most of the time” practicing the behavior at the baseline, but “Occasionally” or “Never” at the follow-up survey was regarded as exhibiting behavioral “fatigue” (see Table 2). Self-reported adherence to COVID preventive measures has been used as a proxy measure of behavioral fatigue [32].

**Table 1:** Perceived infectivity and severity of COVID-19 and perceived benefits of preventive behaviors at baseline and follow-up (n = 403)

	Very High: 5	High: 4	Medium: 3	Low: 2	Very Low: 1	Mean Score	t-test p-value
Perceived Transmissibility of COVID-19							
First Wave March 2020	73.2% (295)	22.1% (89)	3.2% (13)	1.0% (4)	0.5% (2)	4.67	p < 0.001
Third Wave December 2020	60.5% (244)	28.0% (113)	8.9% (36)	2.0% (8)	0.5% (2)	4.46	
Perceived Severity of COVID-19							
First Wave March 2020	46.2% (186)	32.0% (129)	17.4% (70)	4.0% (16)	0.5% (2)	4.19	p = 0.027
Third Wave December 2020	54.2% (218)	28.3% (114)	14.4% (58)	2.2% (9)	0.7% (3)	4.33	
Perceived Benefit of preventive behaviors	Mask wearing	Frequent handwashing	Avoiding international travel	Avoiding public places	Avoiding social gatherings		
% Agree that behavior reduces COVID-19 risk							
First Wave March 2020	98.0% (395)	97.0% (391)	93.0% (374)	93.5% (376)	97.0% (391)		
Third Wave December 2020	99.3% (400)	97.0% (390)	93.8% (377)	89.3% (360)	96.8% (390)		
	p = 0.225	p = 0.999	p = 0.776	p = 0.045	p = 0.999		

**Table 2:** Preventive behaviors against COVID-19 during First and Third waves of Hong Kong pandemic (n = 403)

	Practiced to the highest level possible/ At every opportunity (1 point)	Practiced most of the time/Usually performed (2 points)	Practice Occasionally performed (3 points)	Never/Same as pre-pandemic levels (4 points)	Mean score	t test p-value	Behavioral Fatigue % (n)
**Hygiene practices**							0.0% (0)
Wear mask when going out						< 0.001	
Baseline	93.8% (378)	2.7% (11)	2.7% (11)	0.7% (3)	1.10		
9-month follow-up	98.3% (396)	1.7% (7)	0.0% (0)	0.0% (0)	1.02		
Handwashing with soap or alcohol rubs						0.958	4.7% (19)
Baseline	71.7% (289)	20.3% (82)	6.9% (28)	1.0% (4)	1.37		
9-month follow-up	71.7% (289)	20.3% (82)	6.7% (27)	1.2% (5)	1.37		
**Travel avoidance practices**							
Avoidance of international travel						0.013	13.6% (55)
Baseline	50.6% (204)	31.3% (126)	12.7% (51)	5.5% (22)	1.73		
9-month follow-up	40.2% (162)	37.2% (150)	15.9% (64)	6.5% (26)	1.89		
**Social distancing practices**							
Avoidance of social gatherings						< 0.001	18.4% (74)
Baseline	40.7% (164)	40.4% (163)	15.6% (63)	3.2% (13)	1.81		
9-month follow-up	18.6% (75)	52.1% (210)	25.6% (103)	3.7% (15)	2.14		
Avoidance of public places/ public transport						< 0.001	30.8% (124)
Baseline	11.2% (45)	41.7% (168)	27.0% (109)	20.1% (81)	2.56		
9-month follow-up	4.2% (17)	23.3% (94)	30.8% (124)	41.7% (168)	3.10		

### Statistical analysis

The characteristics of subjects are summarized with descriptive statistics and Cohen’s W was used to assess differences from the general population. For the comparison between the two waves, sample means were compared via the Student’s t-test and two-proportion z test. Independent associated factors for the continued uptake of various protective behaviors were identified using multiple logistic regression models. In order to ensure the inclusion of important confounders [33], variables with p-values < 0.20 in unadjusted logistic regression were included as candidate variables in a backward multiple logistic regression analysis. The level of statistical significance was set at 0.05. All analyses were performed using IBM SPSS Statistics version 24 [34]. Verbal informed consent was obtained from each of the participant prior to the start of each survey which was approved by Survey and Behavioural Research Ethics Board of the university sponsoring the study.

## Results

### Characteristics of the study sample

A total of 725 individuals were recruited in the baseline, of which 56% (n = 403) responded to follow-up survey. There were no large differences in the demographic attributes of the respondents recruited at baseline and those who were retained in the follow-up 9 months later except that the follow-up sample was comprised of more elderly adults (18.7% at baseline and 22.3% at follow-up). The characteristics of the respondents in this longitudinal study are shown in Table 3. Our study sample was comparable to the population characteristics with respect to age, gender, marital status and area of residence but had higher household income and higher educational attainment than the general population. Nearly all respondents (96.5%) reported that they checked COVID-19 related news. Internet/mobile app (45.2%) or television (42.2%) were the primary sources of COVID-19 news updates.

**Table 3:** Characteristics of the study sample

	Study sample (n = 403)		Hong Kong Census population*	Cohen’s W^c^
	n	%	%	
Gender				0.086
Male	199	49.4%	45.1%	
Female	204	50.6%	54.9%	
Age				0.150
18–24	34	8.4%	9.5%	
25–44	118	29.3%	35.3%	
45–64	161	40.0%	36.8%	
65 or older	90	22.3%	18.4%	
Marital status				0.008
Non-married	162	40.2%	39.9%	
Married	240	59.6%	60.1%	
Residential district ^a^				0.015
Hong Kong Island	69	17.1%	17.2%	
Kowloon	126	31.3%	30.6%	
New Territory	208	51.6%	52.2%	
Education ^a^				0.448
Less than secondary school	40	10.0%	25.7%	
Secondary	167	41.6%	43.7%	
Tertiary level	194	48.4%	30.6%	
Household Income ^b^				0.350
<2000–7999	47	11.9%	15.1%	
8000–19,999	67	16.9%	25.9%	
20,000–39,999	96	24.2%	27.8%	
40,000 or more	186	47.0%	31.2%	
Working status				
Employed at least part-time	244	60.5%	NA	
Housewife	41	10.2%	NA	
FT student	19	4.7%	NA	
Retired/unemployed	99	24.6%	NA	
Main source of pandemic updates				
Internet/mobile phone apps	182	45.2%	NA	
Television	170	42.2%	NA	
Newspapers/Print	18	4.5%	NA	
Radio and others	33	8.2%	NA	

^a^ The Hong Kong population Census data additionally included age 15 to 17 years’ old

^b^ The analysis was conducted with household data

^c^ Cohen’s effect size (small: 0.1; medium: 0.3; large: 0.5)

*2016 Census statistics

#### Changes in the COVID-19-related risk perceptions and the perceived benefits of the various preventive behaviors

Perceptions of the person-to-person transmissibility of COVID-19 and perceived severity of COVID-19 disease in the first and third wave of the local epidemic are shown in Table 1. During the 9 months of follow-up, the perceived transmissibility (as an indicator of perceived susceptibility) had declined during this time. At baseline nearly three-quarters of the respondents believed that the transmissibility of COVID-19 was “very high” and this dropped to 60.5% by the third wave. By contrast, the perceived severity of COVID-19 disease had significantly increased in this period. Examination of the changes in the perceived benefit of various behaviors for reducing COVID-19 infection risk is shown in Table 1. It was noted that for four of the five preventive behaviors (wearing mask, hand hygiene, avoidance of social gatherings, and avoidance of international travel), the overwhelming majority of respondents (> 93.0%) felt these behaviors were beneficial for reducing infection risk at baseline and these proportions did not significantly change during the follow-up period. The perceived benefit of avoiding public places/public transport, however, decreased significantly (93.5–89.3%, p < 0.05) at follow-up.

#### Compliance levels with recommended COVID-19 preventive behaviors at baseline and follow-up

The levels of practicing the preventive measures against COVID-19 in the first and third epidemic wave are shown in Table 2. At the time of the 3rd epidemic wave, the group gatherings in public places were limited to two individuals [35]. Mask-wearing increased between the two time periods whereby practically all respondents wore masks in public. Handwashing behaviors remained high throughout both waves and did not show a statistically significant change. By contrast, the study noted decreased compliance with social distancing and international travel behaviors. Compared to personal and household hygiene, compliance of social distancing in public was lower at the 9-month follow-up despite similar social distancing measure tightening by the government during the baseline and follow-up periods. Nearly one-third of respondents showed fatigue in avoiding public transport and public places; nearly one-fifth demonstrated behavioral fatigue in avoiding social gatherings and over one-eighth of the sample showed behavioral fatigue in avoiding foreign travel. Most notably, by 9-month follow-up, 41.7% of the respondents had returned to pre-COVID levels of using public transport and going to public areas despite rising death tolls and higher daily caseloads.

#### Factors associated with fatigue with preventive behaviors against COVID-19

Table 4 showed the respondent characteristics associated with hand hygiene fatigue and avoidance of international travel fatigue. Since all respondents reported wearing face masks by follow-up, behavioral fatigue was not investigated for mask wearing. Although the univariable analysis showed that employment status and declines in the perceived severity of COVID-19 were significantly associated with hand hygiene fatigue, in the multivariable analysis, only blue-collar workers (OR = 4.71, 95% CI: 1.28–17.31) and retired/unemployed people (OR = 4.00, 95% CI: 1.17–13.65) were noted to have greater hand hygiene fatigue as compared with white collar workers. Declines in perceived severity of COVID-19 was only marginally significant in the multivariable model. None of the factors were noted to be significantly associated with fatigue in avoiding of international travel fatigue but high-income respondents, those who had never undergone the mandatory quarantine for international arrivals and those who demonstrated decline in perceived benefits of travel avoidance showed marginally significant associations (p < 0.10) in the multivariable analysis.

**Table 4:** Factors associated with COVID-19 hand hygiene and avoidance of international travel fatigue

		Hand Hygiene Fatigue					Avoidance of International Travel				
		Fatigue % (n)	Univariable		Multivariable		Fatigue % (n)	Univariable		Multivariable	
			OR (95% CI)	p	OR (95% CI)	p		OR (95% CI)	p	OR (95% CI)	p
**Education**											
Secondary/Tertiary	5.3% (19)		1.00				13.9% (50)	1.00			
Up to Primary	0.0% (0)		0.22 (0.01–3.36)	0.432			12.5% (5)	0.89 (0.33–2.38)	0.814		
**Gender**											
Male	6.0% (12)		1.00				15.1% (30)	1.00			
Female	3.4% (7)		0.55 (0.21–1.44)	0.224			12.3% (25)	0.79 (0.45–1.39)	0.410		
**Age group**											
65+	6.7% (6)		1.00		1.00		10.0% (9)	1.00		1.00	
50–64	4.0% (5)		0.58 (0.17–1.97)	0.386	1.93 (0.23–16.29)	0.545	11.2% (14)	1.14 (0.47–2.75)	0.280	1.02 (0.27–3.86)	0.973
35–49	6.2% (6)		0.92 (0.29–3.97)	0.893	1.54 (0.25–9.39)	0.643	18.6% (18)	2.05 (0.87–4.84)	0.101	1.01 (0.39–2.60)	0.985
18–34	2.2% (2)		0.32 (0.06–1.60)	0.164	3.02 (0.53–17.16)	0.212	15.4% (14)	1.64 (0.67-4.00)	0.779	1.75 (0.73–4.21)	0.214
**Household income**											
<2000–19,999 HKD	5.3% (6)		1.00		1.00		8.8% (10)	1.00		1.00	
20,000–39,999 HKD	7.3% (7)		1.42 (0.46–4.37)	0.192	2.06 (0.63–6.75)	0.233	14.6% (14)	1.78 (0.75–4.20)	0.192	1.69 (0.71–4.02)	0.238
40,000 + HKD	3.2% (6)		0.60 (0.19–1.91)	0.600	1.06 (0.29–3.88)	0.934	16.7% (31)	2.08 (0.98–4.43)	0.057	2.02 (0.94–4.31)	0.071
**Marital status**											
Non-married	3.1% (5)		1.00				11.7% (19)	1.00			
Married	5.8% (14)		1.95 (0.69–5.51)	0.210			15.0% (36)	1.33 (0.73–2.41)	0.350		
**Working status**											
White collar	2.2% (4)		1.00		1.00		15.5% (28)	1.00		1.00	
Blue collar	9.5% (6)		4.66 (1.27–17.09)	0.020	**4.71 (1.28–17.31)**	**0.019**	11.1% (7)	0.68 (0.28–1.65)	0.397	0.88 (0.35–2.22)	0.784
Housewife	2.4% (1)		1.11 (0.12–10.17)	0.929	1.19 (0.13–10.95)	0.878	4.9% (2)	0.28 (0.06–1.12)	0.091	0.40 (0.09–1.82)	0.237
Students	0.0% (0)		-	0.998	-	0.998	21.1% (4)	1.46 (0.45–4.72)	0.530	1.35 (0.41–4.51)	0.624
Unemployed/retired	8.1% (8)		3.89 (1.14–13.26)	0.030	**4.00 (1.17–13.65)**	**0.027**	14.1% (14)	0.90 (0.45–1.80)	0.766	1.37 (0.61–3.07)	0.447
**Household member has a chronic condition?**											
No	4.9% (13)		1.00				14.8% (39)	1.00			
Yes	4.3% (6)		0.87 (0.32–2.34)	0.785			11.5% (16)	0.75 (0.40–1.40)	0.366		
**Perceived Severity of Covid-19 disease**											
Remained same or increased	3.7% (12)		1.00		1.00		12.5% (40)	1.00		1.00	
Declined	8.6% (7)		2.44 (0.93–6.40)	0.071	2.55 (0.95–6.85)	0.064	18.5% (15)	1.60 (0.83–3.06)	0.159	1.35 (0.69–2.65)	0.383
**Perceived infectivity of Covid-19 virus**											
Remained same or increased	4.4% (13)		1.00				13.2% (39)	1.00			
Declined	5.6% (6)		1.29 (0.48–3.49)	0.612			15.0% (16)	1.16 (0.62–2.17)	0.646		
**Believe the behavior is beneficial?**											
Remained same or increased	4.6% (18)		1.00				13.1% (50)	1.00		1.00	
Declined	0.0% (0)		1.07 (0.06–19.1)	0.999			27.8% (5)	2.56 (0.88–7.49)	0.086	2.50 (0.86–7.31)	0.094
**Have enough knowledge to cope with COVID-19**											
Disagree/Neutral	5.6% (11)		1.00				14.4% (28)	1.00			
Agree	3.8% (8)		0.67 (0.26–1.70)	0.398			13.0% (27)	0.89 (0.50–1.57)	0.687		
**Had ever undergone quarantine**											
No	0.0% (0)		1.00				28.6% (4)	1.00		1.00	
Yes	4.9% (19)		0.66 (0.04–11.4)	0.999			13.1% (51)	0.38 (0.11–1.25)	0.110	0.37 (0.11–1.21)	0.100

* A backwards stepwise multiple logistic regression was used to evaluate independent predictors of the fatigue for practice each of the behavior, using variables with p < 0.2 in univariable analysis. Predictors in **bold** were the variables retained in the multivariable model with p < 0.05. For non-significant variables in the multivariable analysis, the odds ratios and 95% CI are shown prior to being dropped from the final model. Haldane-Anscombe correction is applied to zero cell counts for binary predictor

The factors associated with social distancing behavior fatigue are shown in Table 5. Lowered perception of the severity of COVID-19 disease was significantly associated with both types of social distancing behavior fatigue (avoidance of social gathering OR = 2.73, 95% CI: 1.54–4.85; avoidance of public areas/public transport OR = 1.69, 95% CI: 1.02–2.82). It was further noted that people with lower education level (OR = 3.65, 95% CI: 1.56–8.57) and those in the highest income group (OR = 2.16, 95% CI: 1.02–4.56) were more likely to report fatigue in the avoidance of social gatherings in the multivariable analysis. Moreover, being non-married was marginally significantly (p = 0.067) associated with avoidance of social gathering fatigue. But lowered perceptions of the transmissibility of COVID-19 and lower perceptions about the benefits of these social distancing behaviors were not independently associated with fatigue of either type of social distancing behavior.

**Table 5:** Factors associated with COVID-19 social distancing behaviors fatigue

	Avoidance of social gathering fatigue					Avoidance of public areas/public transport Fatigue				
	Fatigue % (n)	Univariable		Multivariable		Fatigue % (n)	Univariable		Multivariable	
		OR (95% CI)	p	OR (95% CI)	p		OR (95% CI)	p	OR (95% CI)	p
**Education**										
Secondary/Tertiary	16.9% (61)	1.00		1.00		30.7% (111)	1.00			
Up to Primary	32.5% (13)	2.37 (1.16–4.85)	0.018	**3.65 (1.56–8.57)**	**0.003**	32.5% (13)	1.08 (0.54–2.18)	0.820		
**Gender**										
Male	17.1% (34)	1.00				26.6% (53)	1.00		1.00	
Female	19.6% (40)	1.18 (0.71–1.96)	0.513			34.8% (71)	1.47 (0.96–2.25)	0.076	1.49 (0.97–2.30)	0.071
**Age group**										
65+	16.7% (15)	1.00				25.6% (23)	1.00		1.00	
50–64	16.0% (20)	0.95 (0.46–1.98)	0.896			25.6% (32)	1.00 (0.54–1.87)	0.994	0.60 (0.23–1.57)	0.293
35–49	17.5% (17)	1.06 (0.50–2.28)	0.876			34.0% (33)	1.50 (0.80–2.83)	0.208	0.76 (0.38–1.52)	0.433
18–34	24.2% (22)	1.59 (0.77–3.32)	0.213			39.6% (36)	1.91 (1.01–3.59)	0.046	1.04 (0.54–2.03)	0.899
**Household income**										
<2000–19,999 HKD	14.0% (16)	1.00		1.00		27.2% (31)	1.00		1.00	
20,000–39,999 HKD	17.7% (17)	1.32 (0.63–2.77)	0.467	1.77 (0.78–4.01)	0.175	26.0% (25)	0.94 (0.51–1.74)	0.851	0.87 (0.45–1.68)	0.672
40,000 + HKD	21.0% (39)	1.63 (0.86–3.07)	0.134	**2.16 (1.02–4.56)**	**0.044**	34.9% (65)	1.44 (0.86–2.40)	0.163	1.23 (0.66–2.27)	0.520
**Marital status**										
Non-married	22.2% (36)	1.00		1.00		32.7% (53)	1.00			
Married	15.8% (38)	0.66 (0.40–1.09)	0.106	0.61 (0.35–1.04)	0.067	29.6% (71)	0.86 (0.56–1.33)	0.505		
**Working status**										
White collar	19.9% (36)	1.00				33.1% (60)	1.00		1.00	
Blue collar	17.5% (11)	0.85 (0.40–1.80)	0.674			23.8% (15)	0.63 (0.33–1.22)	0.169	0.62 (0.31–1.22)	0.163
Housewife	14.6% (6)	0.69 (0.27–1.77)	0.440			24.4% (10)	0.65 (0.30–1.42)	0.278	0.52 (0.23–1.21)	0.127
Students	21.1% (4)	1.07 (0.34–3.43)	0.904			47.4% (9)	1.82 (0.70–4.70)	0.220	1.99 (0.76–5.26)	0.164
Unemployed/retired	17.2% (17)	0.84 (0.44–1.58)	0.579			30.3% (30)	0.88 (0.52–1.49)	0.626	0.96 (0.56–1.67)	0.892
**Household member has a chronic condition?**										
No	19.7% (52)	1.00				33.3% (88)	1.00		1.00	
Yes	15.8% (22)	0.77 (0.44–1.33)	0.341			25.9% (36)	0.70 (0.44–1.11)	0.125	0.70 (0.43–1.13)	0.139
**Perceived Severity of Covid-19 disease**										
Remained same or increased	15.3% (49)	1.00		1.00		28.3% (91)	1.00		1.00	
Declined	30.9% (25)	2.48 (1.41–4.34)	0.002	**2.59 (1.45–4.62)**	**0.001**	40.7% (33)	1.74 (1.05–2.88)	0.032	**1.69 (1.02–2.82)**	**0.043**
**Perceived transmissibility of Covid-19 virus**										
Remained same or increased	15.9% (47)	1.00		1.00		29.7% (88)	1.00			
Declined	25.2% (27)	1.79 (1.05–3.06)	0.034	1.45 (0.81–2.60)	0.209	33.6% (36)	1.20 (0.75–1.92)	0.452		
**Believe the behavior is beneficial?**										
Remained same or increased	17.8% (70)	1.00		1.00		30.6% (114)	1.00			
Declined	40.0% (4)	3.08 (0.85–11.19)	0.088	2.90 (0.55–9.46)	0.253	30.0% (9)	0.97 (0.43–2.18)	0.941		
**Have enough knowledge to cope with COVID-19**										
Disagree/Neutral	19.0% (37)	1.00				32.8% (64)	1.00			
Agree	17.8% (37)	0.92 (0.56–1.53)	0.759			28.8% (60)	0.83 (0.54–1.27)	0.388		
**Had ever undergone quarantine**										
No	35.7% (5)	1.00		1.00		50.0% (7)	1.00		1.00	
Yes	17.7% (69)	0.39 (0.13–1.19)	0.099	0.44 (0.13–1.47)	0.184	30.1% (117)	0.43 (0.15–1.25)	0.122	0.56 (0.18–1.69)	0.299

* A backwards stepwise multiple logistic regression was used to evaluate independent predictors of the fatigue for practice each of the behavior, using variables with p < 0.2 in univariable analysis. Predictors in **bold** were the variables retained in the multivariable model with p < 0.05. For non-significant variables in the multivariable analysis, the odds ratios and 95% CI are shown prior to being dropped from the final model. Haldane-Anscombe correction is applied to zero cell counts for binary predictor

## Discussion

This follow-up study noted that in Hong Kong, a region with repeated experience with pandemics such as SARS and avian influenza, personal hygiene measures against COVID-19 were quickly adopted and remained high for almost a year, while adherence to various social distancing measures such as avoidance of going to public places and avoidance of using public transport had significantly declined in the follow-up period. Similarly, avoidance of international travel also declined during this period.

During the 2003 SARS epidemic, the majority of residents of Hong Kong had adopted mask-wearing and personal hygiene measures as a means of self-protection against respiratory diseases. Unlike many other parts of the world, in which face mask use during epidemics has been a political issue [36–39], mask wearing is a well-accepted infection control measure in Hong Kong and is seen as a normative behavior. In our study, this is evidenced by the fact that 100% of the respondents reported that they wore a mask by the second wave to the local epidemic. Moreover, hand hygiene has been encouraged at restaurants, shopping malls, residential buildings and government buildings having alcohol hand sanitizer dispensing stations in the entryways, exits, bathroom facilities in elevator lobbies. The high visibility and accessibility of hand sanitizing dispensers conveniently located in public places could have helped to maintain high levels of hand hygiene [40]. Compared to avoidance on social gathering and travel, mask wearing and hand hygiene behaviors entail less social and emotional cost, they remained high throughout the study period. Unlike personal hygiene measures, social distancing may be very disruptive for work, personal obligations and social life. Moreover, a study [41] showed that living in crowded households is associated with decreased well-being during the COVID-19 pandemic, and due to the fact that the living quarters in Hong Kong are among the smallest in the world with families often residing in apartments that are under 500 square feet [42], socializing is difficult to be done in the home in Hong Kong. Hence, avoidance of public places may have greater effects on social life in Hong Kong than in other areas of the world. Moreover, social distancing is a novel behavior which was not mandated in previous local epidemics which simply called for greater hygiene and sanitation. These factors may partially explain the higher behavioral fatigue in adhering to social distancing measures in this longitudinal study. While the perceived benefits of most preventive behaviors remained very high, over the follow-up period the perceived benefits of avoiding public places declined significantly.

Although hand hygiene behaviors remained high throughout the study period, it was noted that certain occupational groups were significantly more likely to report declining adherence to hand hygiene. The hand hygiene fatigue reported by retired/unemployed individuals may be due to the lower need to leave their home as compared to working people or people with small children. A previous study suggested that perceived personal risk of infection and health effects are linked to engagement in protective behaviors, but individuals are often poor at perceiving risk unless they are equipped with the pertinent knowledge and health information about the epidemic [43]. Since retired people are largely comprised of elderly who are at higher risk of developing severe disease from COVID-19, personal hygiene behaviors should still be promoted even for those who stay at home. This is particularly important as elderly with non-communicable diseases experienced limited access to essential medicines and services during lockdowns [22]. On the other hand, blue-collar workers who reported significant declines in hand hygiene behaviors may be due to several possibilities. Those engaged in food and beverage work may already have had high levels of hand hygiene behaviors due to their jobs at baseline which may have increased during the early stages of the pandemic and returned to baseline levels soon after. It may also reflect lower health consciousness. The current study noted that blue collar worker and people with low education attainment were half as likely to practice dining hygiene behaviors that have been promoted by the government since the 2003 SARS epidemic. In addition to targeted health education, blue collar workers may require provision of hand sanitizers in their workplace and improved hygiene facilities. Past studies of poultry workers in Hong Kong have noted that reducing interference with work was associated with greater adoption of avian influenza preventive behaviors [44]. Additionally, the employers of blue-collar workers may need to have greater accountability during government inspections and in follow-up regulatory actions. Infection disease control efforts should be prioritized to these groups.

Most notable, lower perception of the severity of COVID-19 was associated with social distancing behavior fatigue. Although the daily caseloads of COVID-19 were rising in Hong Kong and in the world, as the pandemic progressed, the case-fatality of the disease began to decrease [45]. During the follow-up period in December 2020, Hong Kong’s case fatality rate was around 1.6% which was lower than many other countries at that time [46]. The level of compliance of these measures relies on individuals weighing the perceived risks associated with the virus versus the costs of adhering to preventive practices. For both types of social distancing behaviors, declines in the perceived benefits of these preventive behaviors and perceptions of the transmissibility of the virus were not associated with behavioral fatigue. In this study, those with lower education were more likely to report social gathering fatigue, suggesting that lower levels of knowledge may contribute to behavioral fatigue. It was also noted in a previous serial cross-sectional study, lower awareness was likely the main reason of lower adherence of social distancing [7]. These findings indicate that health education may need to be targeted to these population subgroups. Studies from the Asia region and the West have noted that higher COVID-related knowledge and health literacy were associated with greater adherence to various preventive behaviors [47–50]. It was also noted that high income respondents reported significantly higher social gathering avoidance fatigue. This may reflect the likelihood that higher income workers may be more likely to be working from home than lower income workers. In a previous study, it also was noted that affluent individuals who were able to pay the additional delivery/minimum order fees for food delivery services were much less likely to eat outside the home during the pandemic [17]. The prolonged social isolation from working and dining at home since the early phase of the pandemic may explain their highly likelihood of subsequent fatigue.

In light of the study’s findings, the role of public health measures is to encourage rapid uptake and long-term maintenance of preventive behaviors in a pandemic. Given that the population reported social distancing and travel avoidance fatigue, measures that encourage continued social distancing need to be reinforced. Since over 90% of the population of Hong Kong commutes via an extensive mass transit system [51], continued vigilance should also be encouraged on public transportation. For instance, from July 2020 onwards, the Hong Kong government prohibited evening dine-in services at restaurants. The government should thereby provide greater support for the increasing demand for food delivery services to help curb infections. Declining avoidance of international travel may partly be addressed by opening travel bubbles with low-risk regions. In order to maintain preventive behaviors long-term, moderate public health actions (reduced seating in restaurants, reduced restaurant service hours) are likely to be much more sustainable than draconian measures (e.g. total lockdowns).

The main strengths of this study are the generally representative study sample and the examination of important preventive behaviors over a long-term follow-up and inclusion of a wide array of potential confounding variables. Yet, this study has several limitations. Firstly, although the land-based telephone list covered more than 85% of land-based telephones in Hong Kong [52], the households that were not on the list of land-based telephone service were not included. Secondly, our study population is older than the general population of Hong Kong. Older individuals are less likely to travel overseas and this may have led to slight underestimations of the avoidance of international travel during the pandemic from baseline levels. Regardless, our sample was comparable with the latest population Census in terms of gender, marital status and area of residence. Thirdly, surveys collected self-reported data may be subject to reporting bias and can only provide a snapshot of the population. Fourthly, we only measured self-reported adherence and did not directly measure the preventive behaviors. In order to obtain more detailed information about risk perceptions and the reasons for behavioral fatigue, qualitative studies may be conducted in the future. Lastly, this study examined Chinese-speaking adults which likely excluded the large numbers of non-Chinese residents of Hong Kong. Notably, Southeast Asian domestic helpers who are given only one rest day per week may experience more fatigue from social distancing measures. Also, foreign workers who come from countries with limited social distancing measures in place may also feel greater fatigue. Future studies could further examine these cultural differences.

## Conclusion

As a likely result of Hong Kong’s experience with successive worldwide pandemics such as SARS, personal hygiene preventive measures against COVID-19 were quickly adopted and maintained by the Hong Kong population. Yet, similar to other countries, the practice of social distancing noticeably declined over this 9-month follow-up period indicating that social distancing will be the key challenge for COVID-19 infection control in a protracted pandemic. Other parts of the world that experience future pandemics may also have similar patterns of preventive behaviors whereby personal protection behaviors will be more readily adopted and maintained than social distancing behaviors. Given this, government efforts will need to be directed towards maintaining social distancing during protracted pandemics, particularly in the workplace and in public areas such as mass transport. Individuals who are noted to be prone to greater pandemic fatigue in other regions (e.g. young people) require targeted efforts to emphasize the potential severity of COVID-19 disease. Health education programs aimed at improving awareness of the potential for severe COVID-19 disease may be helpful to improve continued adherence to those targeted groups. The most appropriate use of various public health and social measures in the context of COVID-19 pandemic across different countries around the world warrants further research [53].

## Data Availability

The datasets used and/or analysed during the current study are available from the corresponding author on reasonable request.
